# A “Much Nervous” Case1Read before the Buffalo Clinical Society, April 9, 1892.

**Published:** 1892-05

**Authors:** William C. Krauss

**Affiliations:** Buffalo, N. Y.


### A "MUCH NERVOUS" CASE.[1]

BY WILLIAM C. KRAUSS, M. D., Buffalo, N. Y.

I BEG the members of this society to bear with the title of my paper until after its reading; then if any apology is necessary on my part, I will unhesitatingly comply. Moreover, my title stands as a challenge for any one to produce a more nervous case, but I hope, for humanity's sake, that none will be forthcoming.

The patient was referred to me by Dr. F., who desired to know what form of treatment would give the most relief.

Name, A. H.; female; *single;* occupation, seamstress; *age, forty-three;* height, four feet ten inches; weight, ninety-eight pounds; complexion, fair; hair, brown; constitution, frail and delicate; temperament, decidedly neurotic.

*Antecedents.*—She has no knowledge of her grandparents on the paternal side, while on the maternal side they reached old age, dying at ninety and eighty-four years respectively. Her father died of tuberculosis when sixty-eight years of age; her mother, still living, is of a highly nervous temperament, but not afflicted with any recognized nervous disease. The aunts and uncles on her mother's side are all subject to heart disease. She had one sister present at the examination, who suffers much from nervous headaches, is extremely irritable, and, in her own words, is "very nervous." One brother, a typesetter, belongs to the same category.

*History.*—When three years old she passed through a severe attack of scarlet fever, and at seven the whooping cough. She made good recoveries without any disagreeable sequelæ. At thirteen she was first afflicted with asthma, the paroxysms becoming very severe at times. At fourteen menstruation appeared, bringing with it a train of amenorrheic symptoms, compelling her to give up her schooling almost entirely.

1. Read before the Buffalo Clinical Society, April 9, 1892.

At sixteen, while watching her companions at play, she was forcibly pulled backwards, receiving an injury to her spine. She complained much of soreness and pain in her back, and was hardly able to walk or move about. Her general condition was very poor at this time and constantly growing worse until her twenty-third year, when she was obliged to keep her bed altogether. Symptoms of myelitis were present at this time, such as spasticity and sensory disturbances of the lower extremities and suppression of urine. To these symptoms were added new ones, more or less hysterical in nature—among others, convulsions, especially after excitement, etc. These attacks were of such frequent occurrence that she decided to try the hot pack treatment, which gave her considerable relief for a short time. Just as she was getting accustomed to bear her loads patiently, she noticed her neck swelling, her eyes bulging, and she experienced attacks of palpitation and flushings. These symptoms of exophthalmic goitre continued in their way for two years, the neck gradually growing larger. In 1880, her left arm began to trouble her. Sharp, shooting pains were felt extending from the shoulder to the tips of the fingers; at the same time it grew weaker and weaker until it was rendered almost powerless. In 1882, the right arm became similarly affected, though to a less degree. After a period of a few months the pain and weakness in the arms would diminish and she would regain partial control over them. Since 1880, the left arm has been helpless ten times, the right three times. In 1886, she had two severe attacks of gastralgia and cardialgia, and her life seemed to be in imminent peril. She rallied, however, and a short time thereafter underwent an operation for the removal of uterine polypi. Of late she has had no recurrence of the asthma.

*Examination.*—The patient, accompanied by her sister, was barely able to come to my office on account of the weakness of her limbs and shuffling gait. I found her a small, sickly-looking woman, possessed of a fair amount of intelligence, extremely anxious to secure some relief from her sufferings.

Her head, rather small, was well formed and regular, and disclosed neither scar nor deformity. The eyes were prominent, pupils symmetrical, field of vision normal on the left side, narrowed and amaurotic on the right. No actromatopsia. Sense of smell was diminished on the right side, sense of hearing also diminished on this side, while the sense of taste was unimpaired. She has been troubled many years with an obstinate nasal catarrh, and has suffered much from earache. She complained of difficulty in swallowing, hoarseness, and a spasmodic cough, all due, no doubt, to the pressure of the goitre upon the larynx. Her neck was markedly increased in size, measuring thirteen and three-quarter inches in circumference. The left lobe of the thyroid gland was larger than the right, and extended well into the left supraclavicular region. The right lobe, though somewhat smaller, extended into the right supraclavicular region. The gland was hard, firm, and dense, growing somewhat smaller and softer towards evening. On auscultation a bruit could be distinctly heard. She complained of sharp, shooting pains, especially on pressure over Erb's point on both sides, radiating to the tips of the fingers, to the shoulder, and along the occiput to the upper level of the ears. The muscles of the right arm were much atrophied, particularly those of the forearm and hand. The muscular force, as measured with the dynamometer, showed ten pounds. Trophic disturbances, such as hyperidrosis, glossy skin, etc., and disturbances of sensation, were also present. The left arm was even more affected than the right. The atrophy of the muscles was far advanced, the dynamometer giving but three pounds pressure. Trophic and sensory disturbances were also present similar to those on the right side. Pressure over the nerve trunks was exceedingly painful, and, when persisted in, the arms were thrown into a series of clonic contractions. The legs were weakened, paretic, the muscles soft and atrophied, the patellar reflexes exaggerated, ankle clonus present on both sides, and the gait spastic. The vesical reflex was impaired, retention of urine being the rule. The urine contained neither albumin nor sugar. The rectal reflex was normal. The general sensibility of the legs was somewhat blunted, trophic disturbances were wanting. An electrical examination of the arms and legs, for obvious reasons, could not be made. The body presented no noteworthy malformation. The spine was painful and tender to the touch, especially between the fourth and tenth dorsal vertebræ. The heart, somewhat enlarged, was rapid, pulse ninety-six, prone to palpitation, and on auscultation revealed a mitral engurgitant murmur. The other internal organs, as far as could be ascertained, were apparently healthy.

The history and examination of such a case furnishes almost material enough for a first-class clinic throughout a good part of the college year. Perhaps her occupation as seamstress is responsible for the snarl which the threads of disease have produced! To unravel it was a task by no means as easy as it may appear.

Her family history on the mother's side savors of a neurotic tendency, while on her father's side there appears to be a tubercular diathesis. I know of no combination so favorable for the transmission of hereditary disease. This is, then, briefly a story of her life. Scarlet fever at three, whooping cough at seven, asthma at fourteen, myelitis at tweny-three, exophthalmic goitre at thirty-five, pressure neuritis with paresis of the upper extremities at thirty-seven, gastralgia and cardialgia at thirty-nine, and hysteria dating back to the beginning of her menstrual periods. To these must be added such minor affections as amenorrhea, chronic nasal and aural catarrh, mitral disease and uterine polypi.

I advised a partial thyroidectomy as affording the most immediate relief, believing that the neuritis was dependent upon pressure of the goitre upon the cervical and brachial plexuses at their points of origin.

